# Crystal engineering with short-chained amphiphiles: deca­sodium octa-*n*-butane­sulfonate di-μ-chlorido-bis­[di­chlorido­palladate(II)] tetra­hydrate, a layered inorganic–organic hybrid material

**DOI:** 10.1107/S2056989019004201

**Published:** 2019-04-02

**Authors:** Felix Thoelen, Walter Frank

**Affiliations:** a Institut für Anorganische Chemie und Strukturchemie, Lehrstuhl II: Material- und Strukturforschung, Heinrich-Heine-Universität Düsseldorf, D-40225 Düsseldorf, Germany

**Keywords:** crystal structure, sodium *n*-butane­sulfonate, hexa­chlorido­dipalladate(II), amphiphile, bilayered structure, inorganic–organic hybrid material

## Abstract

In the course of crystal-engineering experiments, crystals of the hydrated title salt, Na_10_[Pd_2_Cl_6_](C_4_H_9_SO_3_)_8_·4H_2_O, were obtained from a water/2-propanol solution of sodium *n*-butane­sulfonate and sodium tetra­chlorido­palladate(II). In the crystal, sodium *n*-butane­sulfonate anions and water mol­ecules are arranged in an amphiphilic inverse bilayered cationic array represented by the formula {[Na_10_(C_4_H_9_SO_3_)_8_(H_2_O)_4_]^2+^}_*n*_. Within this lamellar array: (i) a hydro­philic layer region parallel to the *bc* plane is established by the Na^+^ cations, the H_2_O mol­ecules (as aqua ligands in κ*Na*,κ*Na*′-bridging coordination mode) and the O_3_S– groups of the sulfonate ions, and (ii) hydro­phobic regions are present containing all the *n*-butyl groups in an almost parallel orientation, with the chain direction approximately perpendicular to the aforementioned hydro­philic layer. Unexpectedly, the flat centrosymmetric [Pd_2_Cl_6_]^2−^ anion in the structure is placed between the butyl groups, *within* the hydro­phobic regions, but due to its appropriate length primarily bonded to the hydro­philic ‘inorganic’ layer regions above and below the hydro­phobic area *via* Pd—Cl_t_⋯Na- and Pd—Cl_t_⋯H—O(H)—Na-type (Cl_t_ is terminal chloride) inter­actions. In addition to these hydrogen-bonding inter­actions, both aqua ligands are engaged in charge-supported S—O⋯H—O hydrogen bonds of a motif characterized by the *D*_4_^3^(9) graph-set descriptor within the hydro­philic region. The crystal structure of the title compound is the first reported for a metal *n*-butane­sulfonate.

## Abstract 2

The preparation and crystal structure of the layered inorganic–organic hybrid material are presented. The crystal structure determination of Na_10_(C_4_H_9_SO_3_)_8_[Pd_2_Cl_6_]·4H_2_O is the first of a metal *n*-butane­sulfonate and confirms a unique lamellar amphiphilic bilayered structure with the hexa­chlorido­dipalladate(II) ions unexpectedly placed within the ‘organic’ hydro­phobic layer region but primarily bonded to the neighbouring ‘inorganic’ hydro­philic layers *via* hydrogen bonding and ‘local’ ionic inter­actions.

## Chemical context   

Sodium alkane­sulfonates are artificial soaps (anionic tensides) with a widespread use (Schramm *et al.*, 2003 ▸). They are known to have a bilayered structure like ‘natural’ soaps, with an extreme tendency for disorder in the crystalline state (Buerger, 1942 ▸; Buerger *et al.*, 1942 ▸). Compounds containing alkane­sulfonate ions of the general formula C_*n*_H_2*n*+1_SO_3_^−^ with *n* = 1–4 may be defined as short-chained alkane­sulfonates (SCAS). In contrast to methane­sulfonates (*n* = 1) and ethane­sulfonates (*n* = 2), there is only rare structure information for the next higher homologues (*n* = 3, 4) (Frank & Jablonka, 2008 ▸; Russell *et al.*, 1994 ▸). Solid sodium methane­sulfonate is described as an inorganic–organic three-dimensional network (Wei & Hingerty, 1981 ▸). However, closer inspection shows the compound to have a bilayered soap-like structure with only one of five CH_3_SO_3_^−^ anions connecting in the third dimension. In crystal-engineering experiments, we successfully exchanged this connecting anion by selected other ionic moieties and were able to retain the lamellar structure (Thoelen & Frank, 2017 ▸, 2018 ▸; Verheyen & Frank, 2009 ▸). An aim of subsequent attempts was to include chlorido­palladate(II) anions Pd_*n*_Cl_2*n*+2_^2−^ that are known to be catalytically active (Bouquillion *et al.*, 1999 ▸; Jimeno *et al.*, 2012 ▸; Lassahn *et al.*, 2003 ▸; Mu *et al.*, 2012 ▸), by using [PdCl_4_]^2−^ in the form of its sodium salt as a typical precursor in aqueous palladium(II) chemistry.
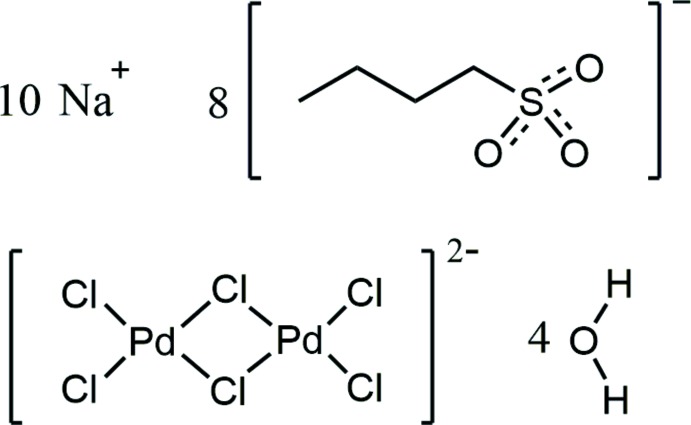


In the investigation described herein, the incorporation of hexa­chlorido­dipalladate(II) anions into the sodium *n*-butane­sulfonate layered system was realized, resulting in the title compound (**1**) having the typical brown colour of palladium complexes with a square-planar coordination environment. According to the results of elemental analysis and vibrational spectroscopic investigations, hydrated sodium cations, *n*-butane­sulfonate and hexa­chlorido­dipalladate(II) anions are present in the solid. The crystal structure determination of this compound is the first of a metal *n*-butane­sulfonate and eventually confirmed the composition Na_10_(C_4_H_9_SO_3_)_8_[Pd_2_Cl_6_]·4H_2_O and a lamellar amphiphilic structure.

## Structural commentary   

**Figure 1:**
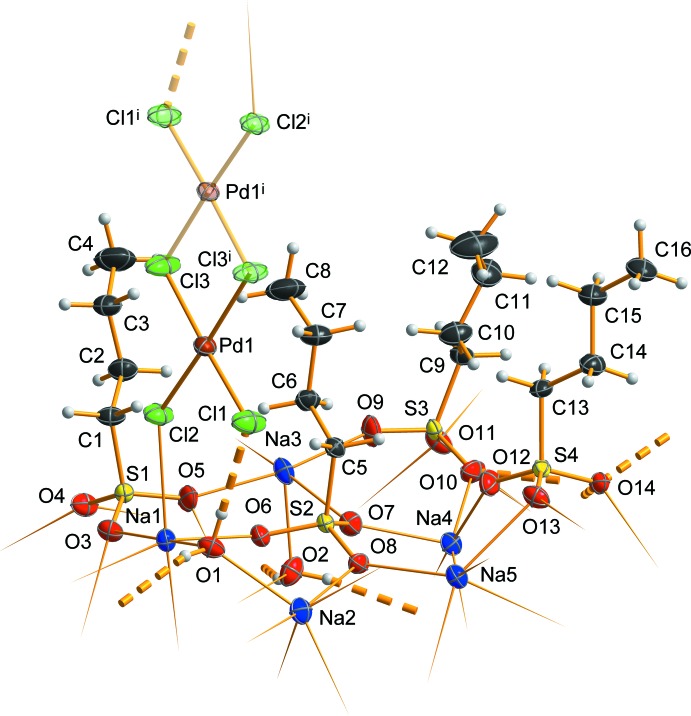
The asymmetric unit of **1**, chosen to give a compact segment with all *n*-butyl groups of the hydro­phobic layer region oriented in one direction. In addition, the symmetry-related second half of the hexa­chlorido­dipallate(II) anion is shown in transparent mode [symmetry code: (i) −*x*, 1 − *y*, 1 − *z*.]. The direction of coordinative bonding to atoms of neighbouring moieties is given by sharpened sticks, and hydrogen bonds are shown as segmented solid bonds. Displacement ellipsoids are drawn at the 50% probability level, hydrogen atoms are drawn with an arbitrary radius. Note the coordination of the hexa­chlorido­dipalladate(II) ion to hydro­philic moieties by hydrogen bonding and ‘local’ ionic inter­actions.

**Figure 2:**
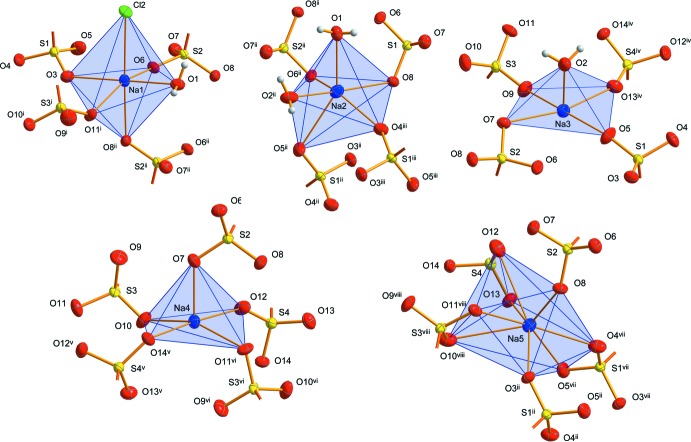
Coordination environments of sodium cations. For clarity, *n*-butyl­groups of the *n*-butane­sulfonate anions are not shown. [Symmetry codes: (i) *x*, 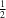 − *y*, 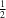 + *z*; (ii) 1 − *x*, 1 − *y*, 1 − *z*; (iii) *x*, 1 + *y*, *z*; (iv) *x*, −1 + *y*, *z*; (v) 1 − *x*, −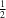 + *y*, 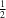 − *z*; (vi) 1 − *x*, 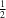 + *y*, 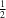 − *z*]. The Na—O distances [Na1—O = 2.284 (3)–2.540 (3) Å; Na2—O = 2.283 (3)–2.700 (3) Å; Na3—O = 2.212 (2)–2.649 (3) Å; Na4—O = 2.308 (5)–2.479 (3) Å; Na5—O = 2.391 (3)–3.000 (4) Å] are within the reported range for short-chained sodium alkane­sulfonates (Wei & Hingerty, 1981 ▸).

**Figure 3:**
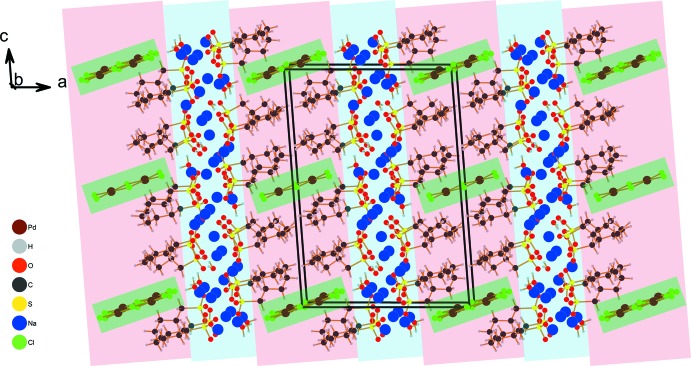
Diagram displaying hydro­philic (blue) and hydro­phobic sections (red) of the bilayered amphiphile packing of **1**; layers are parallel to the *bc* plane of the unit cell. Note the inverse bilayer thickness corresponding to the unit-cell dimension along [100]. The hexa­chlorido­dipalladate(II) anions are placed within the hydro­phobic region but are primarily bonded to the hydro­philic.

Fig. 1 ▸ shows the asymmetric unit of the crystal structure that contains (all in general positions) five sodium cations, two water mol­ecules, four *n*-butane­sulfonate anions and, close to a center of inversion, one half of a hexa­chlorido­dipalladate anion. The five Na^+^ cations are in quite different coordination environments (Fig. 2 ▸), defined by five sulfonato ligands (Na4, Na5), four sulfonato ligands and one aqua ligand (Na3), four sulfonato ligands and two aqua ligands (Na2) and four sulfon­ato ligands, one aqua ligand and one terminal chlorido ligand of the [Pd_2_Cl_6_]^2−^ anion (Na1). Bond lengths and angles of the *n*-butanesulfonate anions are as expected (see supplementary Tables). All these anions are found with an entirely *anti*-periplanar conformation of the alkyl groups, without any disorder. Altogether, *n*-butane­sulfonate anions, Na^+^ cations and water mol­ecules form a tenside-like inverse bilayered cationic array, which can be described by the formula {[Na_10_(H_2_O)_4_(C_4_H_9_SO_3_)_8_]^2+^}_*n*_. In this arrangement, the layer-like regions are oriented parallel to the *bc* plane of the unit cell. As visualized by the blue and the red sections of the transparent background of Fig. 3 ▸, hydro­philic and hydro­phobic regions are given, reminiscent of sections of the structures of ‘pure’ short-chained sodium alkane­sulfonates (Frank & Jablonka, 2008 ▸; Wei & Hingerty, 1981 ▸). The hydro­philic areas contain the Na^+^ cations, the H_2_O mol­ecules serving as aqua ligands in *μ*(κNa,κNa′) bridging mode coordination, and the O_3_S– groups of the sulfonate ions. With all the C_4_-chains in an approximately parallel orientation, the butyl groups are arranged on both sides of the hydro­philic region to complete the amphiphilic double layer with an inverse bilayer thickness according to unit-cell parameter *a*. The centrosymmetric [Pd_2_Cl_6_]^2−^ anions in the structure of **1** are placed between the *n*-butyl groups *within the hydro­phobic regions*. In a first view, this position seems to be unexpected; however, the length of the dipalladate(II) anion is appropriate to allow for pronounced bonding to the hydro­philic ‘inorganic’ layered regions above and below the hydro­phobic area (Fig. 3 ▸). To inter­act with the inorganic areas above and below the hydro­phobic region, a building block is needed that fits to the thickness of the hydro­phobic double layer. In the concrete case of **1**, the thickness is determined by the lengths of two ‘end-facing’ *n*-butyl groups.

As expected, the Pd—Cl bonds to the terminal chlorido ligands [2.2776 (12) and 2.2800 (10) Å] are slightly shorter than the Pd—*μ*-Cl bonds [2.3159 (11) and 2.3212 (12) Å]. These geometric parameters, as well as the Cl—Pd—Cl bond angles of 86.20 (4) to 92.45 (4)° and the Pd—*μ*-Cl—Pd angle of 93.80 (4)°, are in good agreement with those found in Cs_2_[Pd_2_Cl_6_] (Schüpp & Keller, 1999 ▸) or in several hexa­chlorido­dipalladates with large organic cations (*e.g.* Chitanda *et al.*, 2008 ▸; Gerisch *et al.*, 1997 ▸; Makitova *et al.*, 2007 ▸). Alternatively to the formula given above, compound **1** might be formulated as a hydrated double salt of sodium *n*-butane­sulfonate and sodium hexa­chlorido­dipalladate(II): Na_8_(C_4_H_9_SO_3_)_8_·Na_2_Pd_2_Cl_6_·4H_2_O. This choice takes into account that the Na—Cl distance from the terminal chlorido ligand Cl2 of the hexa­chlorido­dipalladate(II) anion to the sodium cation Na1 [2.8560 (18) Å] is close to the distances of 2.809 (3) to 2.821 (2) Å in Na_2_PdCl_4_ (Schröder & Keller, 1989 ▸). However, this is a singular similarity, and because all the sodium cations of **1** clearly are components of the layer-like hydro­philic region, there is a much closer structural relationship of **1** to sodium methane­sulfonate (Wei & Hingerty, 1981 ▸) and sodium 1-propane­sulfonate monohydrate (Frank & Jablonka, 2008 ▸). As in the structures of these compounds, the asymmetric unit in **1** contains five Na^+^ cations, establishing a closely related Na—O coordination network, and the separation of hydro­philic layers and hydro­phobic areas is similar to the most prominent structural feature of crystallized amphiphiles where the neighbouring hydro­phobic areas in the layer-like structures are connected by van der Waals forces only.

## Supra­molecular features   

As emphasized in Fig.1, in addition to the coordinative bonding to two Na^+^ cations [O1—Na1 = 2.326 (3) Å, O1—Na2 = 2.407 (4) Å; O2—Na3 = 2.311 (4) Å, O2—Na2′ = 2.488 (4) Å], the two crystallographically independent water mol­ecules O1 and O2 in **1** are engaged in non-covalent bonding within the hydro­philic region (Table 1 ▸). The water mol­ecule containing O1 serves as donor for both a charge-supported O—H⋯Cl-type hydrogen bond of medium strength to one of the terminal chlorido ligands of the [Pd_2_Cl_6_]^2−^ anion [*D*⋯*A* distance = 3.127 (3) Å] and a charge-supported weak O—H⋯O type hydrogen bond to an O atom of a sulfonate anion containing S4 [*D*⋯*A =* 2.879 (4) Å]. In contrast, the water mol­ecule containing O2 is engaged in two O—H⋯O type hydrogen bonds to sulfonate ions, one of moderate strength to an O atom of the sulfonate ion containing S4 [*D*⋯*A* = 2.723 (4) Å] and a weak one to an O atom of the sulfonate ion containing S3 [*D*⋯*A* = 2.884 (4) Å]. Pd—Cl_term_⋯H—O(Na_2_)—H⋯O14(S4)⋯H—O(Na_2_)—H⋯O—S is the entire path of hydrogen bonding described by the *D* ^3^_4_(9) graph-set descriptor (Russell *et al.*, 1994 ▸; Grell *et al.*, 1999 ▸), with the sulfonate oxygen atom O14 as the central double acceptor.

## Database survey   

A search in the Cambridge Structural Database (Version 5.40, update November 2018; Groom *et al.*, 2016 ▸) for short-chained sodium alkane­sulfonates Na(C_*n*_H_2*n*+1_SO_3_) with *n* = 1–4 gave three hits, *viz.* the structures of sodium methane­sulfonate (BAKLAA; Wei & Hingerty, 1981 ▸), sodium 1-propane­sulfonate monohydrate (GOKHIY; Frank & Jablonka, 2008 ▸) and α-cyclo­dextrin sodium 1-propane­sulfonate nona­hydrate (ACDPRS; Harata, 1977 ▸). For crystal structures with *n*-butane­sulfonate anions, only one entry was found (WETNUE; Russell *et al.*, 1994 ▸), describing the lamellar structure of guanidinium *n*-butane­sulfonate. Searching for the hexa­chlorido­dipalladate(II) anion results in 46 entries. However, from a structural point of view, the role of the [Pd_2_Cl_6_]^2−^ ion in **1** is completely different from the role of this species in all the other compounds. In addition to the reports on these compounds having organic components, there is one report on an inorganic ternary chloride containing the [Pd_2_Cl_6_]^2−^ ion (CsPdCl_3_; Schüpp & Keller, 1999 ▸).

## Synthesis and Crystallization   

Thin brown platelets of **1** were obtained by slow isothermal evaporation of the solvent from a solution of 5 ml of distilled water and 5 ml of iso­propanol containing 3.203 g (20 mmol) of sodium *n*-butane­sulfonate and 1.177 g (4 mmol) of sodium tetra­chlorido­palladate(II). The evaporation temperature of the solution was adjusted to 288 K with a thermostat. After three days, crystals suitable for X-ray crystal structure determination could be harvested (5.985 g; 81.6% based on PdCl_4_^2–^). A single crystal was selected directly from the mother liquor. Raman spectroscopy was done with a Bruker MultiRAM spectrometer, equipped with a Nd:YAG laser (1064 nm) and an InGaAs detector (4000–70 cm^−1^): ν(C—H): 2969 (*m*), 2920 (*s*), 2872 (*m*); δ_s_(C—H): 1445 (*w*), 1412 (*w*); δ_as_(C—H): 1306 (*w*); ν_as_(S—O): 1071 (*s*); ν_s_(C—S): 800 (*m*); δ(S—O): 551 (*m*), 536 (*m*); ν(Pd—Cl_term_): 343 (*m*), ν(Pd—μ-Cl): 305 (*s*); ν(Pd—μ-Cl): 273 (*m*). Band assignments were made according to Fujimori (1959 ▸) and Gerisch *et al.* (1997 ▸). An IR spectrum was recorded by using a Spektrum Two FT–IR spectrometer (Perkin Elmer company) with an LiTaO_3_ detector (4000–350 cm^−1^) and an universal ATR equipment: ν(O—H): 3503 (*s*), 3462 (*sh*), 3436 (*s*), 3367 (s); ν(C—H): 2967 (*s*), 2936 (*s*), 2872 (*m*); δ(O—H): 1662 (*m*), 1602 (*m*); δ_s_(C—H) 1465 (*m*), 1412 (*w*), 1378 (*w*), δ_as_(C—H): 1314 (*w*), 1286 (*w*); ν_as_(C—H): 1241 (*w*); ν_s_(S—O): 1190 (*s*), 1166 (s); ν_as_(S—O): 1057 (*s*), 1044 (*s*); ν_s_(C—S): 794 (*m*); δ(S—O): 555 (*m*), 534 (*m*); band assignment according to Fujimori (1959 ▸). A CHS analysis was performed with a vario micro cube (Elementar Analysensysteme GmbH). Analysis calculated for C_32_H_80_Cl_6_Na_10_O_28_Pd_2_S_8_ (1824.84 g mol^−1^): C 21.06, H 4.42, S 14.06; found: C 20.78, H 4.49, S 12.98.

## Refinement   

Crystal data, data collection and structure refinement details are summarized in Table 2 ▸. The positions of all hydrogen atoms were identified in difference-Fourier syntheses. In the course of the converging refinement, a riding model was applied using idealized C—H bond lengths (0.97–0.98 Å) as well as H—C—H and C—C—H angles. In addition, H atoms of CH_3_ groups were allowed to rotate around the neighboring C—C bonds. The *U*_iso_(H) values were set to 1.5*U*_eq_(C_meth­yl_) and 1.2*U*_eq_(C_methyl­ene_), respectively. H—O distances of the water mol­ecules were restrained to 0.83 (3) Å.

## Supplementary Material

Crystal structure: contains datablock(s) I. DOI: 10.1107/S2056989019004201/wm5491sup1.cif

Structure factors: contains datablock(s) I. DOI: 10.1107/S2056989019004201/wm5491Isup2.hkl

CCDC reference: 1906335

Additional supporting information:  crystallographic information; 3D view; checkCIF report

## Figures and Tables

**Table 1:** Hydrogen-bond geometry (Å, °)

*D*—H⋯*A*	*D*—H	H⋯*A*	*D*⋯*A*	*D*—H⋯*A*
O1—H1⋯O14^i^	0.80 (3)	2.10 (4)	2.879 (4)	164 (5)
O1—H2⋯Cl1	0.82 (3)	2.32 (4)	3.127 (3)	172 (6)
O2—H3⋯O10^ii^	0.81 (3)	1.95 (4)	2.723 (4)	159 (5)
O2—H4⋯O14^ii^	0.80 (3)	2.10 (4)	2.884 (4)	164 (6)

Symmetry codes: (i) 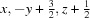; (ii) 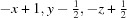.

**Table 2:** Experimental details

Crystal data	
Chemical formula	Na_10_[Pd_2_Cl_6_](C_4_H_9_O_3_S)_8_·4H_2_O
*M*_r_	1824.84
Crystal system, space group	Monoclinic, *P*2_1_/*c*
Temperature (K)	213
*a*, *b*, *c* (Å)	15.9049 (4), 9.9047 (2), 22.6734 (7)
β (°)	94.315 (2)
*V* (Å^3^)	3561.69 (16)
*Z*	2
Radiation type	Mo *K*α
μ (mm^−1^)	1.10
Crystal size (mm)	0.43 × 0.13 × 0.06

Data collection	
Diffractometer	Stoe IPDS_2T
Absorption correction	Multi-scan (*PLATON*; Spek, 2009 ▸)
*T*_min_, *T*_max_	0.650, 0.937
No. of measured, independent and observed [*I* > 2σ(*I*)] reflections	48866, 8183, 7116
*R*_int_	0.072
(sin θ/λ)_max_ (Å^−1^)	0.650

Refinement	
*R*[*F* ^2^ > 2σ(*F* ^2^)], *wR*(*F* ^2^), *S*	0.062, 0.099, 1.54
No. of reflections	8183
No. of parameters	408
No. of restraints	4
H-atom treatment	H atoms treated by a mixture of independent and constrained refinement
Δρ_max_, Δρ_min_ (e Å^−3^)	0.73, −0.43

Computer programs: *X-AREA* (Stoe & Cie, 2009 ▸), *SHELXT* (Sheldrick, 2015*a* ▸), *SHELXL2018* (Sheldrick, 2015*b* ▸), *DIAMOND* (Brandenburg, 2016 ▸) and *publCIF* (Westrip, 2010 ▸).

## supplementary crystallographic information

### Crystal data

**Table:**

Na_10_[Pd_2_Cl_6_](C_4_H_9_O_3_S)_8_·4H_2_O	*F*(000) = 1856
*M**_r_* = 1824.84	*D*_x_ = 1.702 Mg m^−^^3^*D*_m_ = 1.712 Mg m^−^^3^*D*_m_ measured by flotation in chloroform/bromoform
Monoclinic, *P*2_1_/*c*	Mo *K*α radiation, λ = 0.71073 Å
*a* = 15.9049 (4) Å	Cell parameters from 49957 reflections
*b* = 9.9047 (2) Å	θ = 4.1–59.3°
*c* = 22.6734 (7) Å	µ = 1.10 mm^−^^1^
β = 94.315 (2)°	*T* = 213 K
*V* = 3561.69 (16) Å^3^	Thin platelets, brown
*Z* = 2	0.43 × 0.13 × 0.06 mm

### Data collection

**Table:**

Stoe IPDS_2T diffractometer	7116 reflections with *I* > 2σ(*I*)
ω scan	*R*_int_ = 0.072
Absorption correction: multi-scan (*PLATON*; Spek, 2009)	θ_max_ = 27.5°, θ_min_ = 2.1°
*T*_min_ = 0.650, *T*_max_ = 0.937	*h* = −20→20
48866 measured reflections	*k* = −11→12
8183 independent reflections	*l* = −29→29

### Refinement

**Table:**

Refinement on *F*^2^	4 restraints
Least-squares matrix: full	Hydrogen site location: mixed
*R*[*F*^2^ > 2σ(*F*^2^)] = 0.062	H atoms treated by a mixture of independent and constrained refinement
*wR*(*F*^2^) = 0.099	*w* = 1/[σ^2^(*F*_o_^2^) + (0.0215*P*)^2^ + 3.8152*P*] where *P* = (*F*_o_^2^ + 2*F*_c_^2^)/3
*S* = 1.54	(Δ/σ)_max_ < 0.001
8183 reflections	Δρ_max_ = 0.73 e Å^−^^3^
408 parameters	Δρ_min_ = −0.43 e Å^−^^3^

### Special details

**Table:**

Geometry. All esds (except the esd in the dihedral angle between two l.s. planes) are estimated using the full covariance matrix. The cell esds are taken into account individually in the estimation of esds in distances, angles and torsion angles; correlations between esds in cell parameters are only used when they are defined by crystal symmetry. An approximate (isotropic) treatment of cell esds is used for estimating esds involving l.s. planes.

### Fractional atomic coordinates and isotropic or equivalent isotropic displacement parameters (Å^2^)

**Table:**

	*x*	*y*	*z*	*U*_iso_*/*U*_eq_	
Pd1	0.10273 (2)	0.51478 (3)	0.52464 (2)	0.02493 (8)	
Cl1	0.18814 (7)	0.69241 (11)	0.55153 (7)	0.0452 (3)	
Cl2	0.20910 (6)	0.36261 (11)	0.54280 (6)	0.0361 (3)	
Cl3	0.01032 (7)	0.34122 (11)	0.49624 (7)	0.0465 (3)	
S1	0.37572 (5)	0.04330 (8)	0.48827 (4)	0.01605 (17)	
S2	0.40803 (5)	0.51616 (8)	0.41684 (4)	0.01565 (17)	
S3	0.37879 (6)	0.32626 (9)	0.19446 (4)	0.01684 (17)	
S4	0.35920 (5)	0.87006 (9)	0.26546 (4)	0.01710 (17)	
Na1	0.38885 (9)	0.35553 (15)	0.55924 (7)	0.0216 (3)	
Na2	0.46690 (10)	0.73138 (16)	0.54410 (7)	0.0283 (4)	
Na3	0.38987 (10)	0.20007 (16)	0.34308 (7)	0.0274 (3)	
Na4	0.48859 (9)	0.56786 (15)	0.28553 (7)	0.0230 (3)	
Na5	0.46642 (10)	0.86689 (15)	0.39298 (7)	0.0245 (3)	
O1	0.36894 (19)	0.5796 (3)	0.58556 (14)	0.0283 (6)	
H1	0.367 (3)	0.583 (5)	0.6209 (15)	0.037 (15)*	
H2	0.322 (2)	0.604 (6)	0.574 (3)	0.054 (18)*	
O2	0.5354 (2)	0.1883 (3)	0.35206 (15)	0.0329 (7)	
H3	0.556 (3)	0.116 (4)	0.344 (2)	0.040 (15)*	
H4	0.552 (4)	0.240 (5)	0.328 (2)	0.053 (18)*	
O3	0.40321 (17)	0.1195 (3)	0.54127 (12)	0.0241 (6)	
O4	0.40499 (17)	−0.0958 (3)	0.49020 (14)	0.0291 (6)	
O5	0.40033 (19)	0.1094 (3)	0.43503 (13)	0.0334 (7)	
O6	0.41514 (16)	0.4210 (3)	0.46581 (12)	0.0250 (6)	
O7	0.43673 (17)	0.4534 (3)	0.36366 (12)	0.0248 (6)	
O8	0.45103 (16)	0.6439 (3)	0.43105 (12)	0.0232 (6)	
O9	0.3628 (2)	0.2671 (3)	0.25054 (13)	0.0351 (7)	
O10	0.42717 (18)	0.4508 (3)	0.20055 (14)	0.0317 (7)	
O11	0.41987 (17)	0.2306 (3)	0.15672 (12)	0.0268 (6)	
O12	0.38437 (18)	0.7351 (3)	0.28429 (14)	0.0318 (7)	
O13	0.37292 (17)	0.9671 (3)	0.31322 (12)	0.0282 (6)	
O14	0.39915 (16)	0.9112 (3)	0.21230 (11)	0.0227 (6)	
C1	0.2645 (2)	0.0366 (4)	0.48516 (18)	0.0263 (8)	
H1A	0.247524	−0.006022	0.521426	0.032*	
H1B	0.242490	0.129115	0.484160	0.032*	
C2	0.2242 (3)	−0.0399 (5)	0.4324 (2)	0.0322 (9)	
H2A	0.237766	0.005670	0.395932	0.039*	
H2B	0.247891	−0.131170	0.431978	0.039*	
C3	0.1293 (3)	−0.0491 (5)	0.4338 (2)	0.0397 (11)	
H3A	0.115781	−0.088804	0.471540	0.048*	
H3B	0.105327	0.042000	0.431444	0.048*	
C4	0.0894 (4)	−0.1334 (8)	0.3836 (3)	0.0677 (19)	
H4A	0.101675	−0.093553	0.346175	0.102*	
H4B	0.028870	−0.136552	0.386313	0.102*	
H4C	0.112114	−0.224331	0.386268	0.102*	
C5	0.2997 (2)	0.5537 (4)	0.40159 (18)	0.0218 (8)	
H5A	0.276788	0.588722	0.437482	0.026*	
H5B	0.293682	0.624250	0.371248	0.026*	
C6	0.2495 (2)	0.4298 (4)	0.3802 (2)	0.0280 (9)	
H6A	0.272963	0.394351	0.344612	0.034*	
H6B	0.255302	0.359666	0.410749	0.034*	
C7	0.1563 (3)	0.4605 (5)	0.3663 (2)	0.0372 (10)	
H7A	0.149960	0.526981	0.334304	0.045*	
H7B	0.133087	0.499702	0.401316	0.045*	
C8	0.1075 (3)	0.3334 (6)	0.3481 (3)	0.0614 (17)	
H8A	0.048969	0.356374	0.337654	0.092*	
H8B	0.110742	0.269793	0.380703	0.092*	
H8C	0.131600	0.292931	0.314234	0.092*	
C9	0.2808 (2)	0.3675 (4)	0.15778 (17)	0.0248 (8)	
H9A	0.290621	0.413088	0.120555	0.030*	
H9B	0.250007	0.283805	0.147917	0.030*	
C10	0.2260 (3)	0.4574 (5)	0.1931 (2)	0.0374 (11)	
H10A	0.210258	0.408265	0.228162	0.045*	
H10B	0.258374	0.537204	0.206608	0.045*	
C11	0.1472 (3)	0.5017 (6)	0.1572 (3)	0.0472 (13)	
H11A	0.119599	0.421943	0.138975	0.057*	
H11B	0.163221	0.560869	0.125302	0.057*	
C12	0.0847 (3)	0.5754 (6)	0.1927 (4)	0.070 (2)	
H12A	0.040022	0.612826	0.166142	0.105*	
H12B	0.113199	0.647803	0.215028	0.105*	
H12C	0.060712	0.512844	0.219803	0.105*	
C13	0.2498 (2)	0.8633 (4)	0.2452 (2)	0.0265 (8)	
H13A	0.239772	0.797174	0.213276	0.032*	
H13B	0.220841	0.831048	0.279147	0.032*	
C14	0.2112 (2)	0.9972 (4)	0.2249 (2)	0.0315 (9)	
H14A	0.242041	1.032898	0.192421	0.038*	
H14B	0.217053	1.062014	0.257575	0.038*	
C15	0.1180 (3)	0.9829 (5)	0.2040 (2)	0.0379 (10)	
H15A	0.112726	0.920407	0.170397	0.045*	
H15B	0.087968	0.943249	0.235982	0.045*	
C16	0.0765 (3)	1.1155 (5)	0.1856 (2)	0.0432 (12)	
H16A	0.106069	1.155756	0.154136	0.065*	
H16B	0.078677	1.176286	0.219272	0.065*	
H16C	0.018177	1.099456	0.171769	0.065*	

### Atomic displacement parameters (Å^2^)

**Table:**

	*U*^11^	*U*^22^	*U*^33^	*U*^12^	*U*^13^	*U*^23^
Pd1	0.01842 (13)	0.02374 (15)	0.03245 (17)	0.00385 (12)	0.00072 (11)	0.00322 (13)
Cl1	0.0311 (5)	0.0272 (5)	0.0747 (9)	0.0030 (4)	−0.0117 (5)	−0.0018 (6)
Cl2	0.0231 (5)	0.0286 (5)	0.0551 (7)	0.0068 (4)	−0.0055 (5)	0.0019 (5)
Cl3	0.0225 (5)	0.0257 (5)	0.0895 (10)	0.0045 (4)	−0.0078 (6)	0.0017 (6)
S1	0.0189 (4)	0.0142 (4)	0.0150 (4)	−0.0004 (3)	0.0011 (3)	−0.0014 (3)
S2	0.0176 (4)	0.0146 (4)	0.0147 (4)	0.0009 (3)	0.0008 (3)	0.0012 (3)
S3	0.0225 (4)	0.0154 (4)	0.0123 (4)	−0.0003 (3)	−0.0002 (3)	0.0009 (3)
S4	0.0165 (4)	0.0174 (4)	0.0174 (4)	0.0015 (3)	0.0016 (3)	0.0020 (3)
Na1	0.0265 (8)	0.0197 (7)	0.0185 (7)	0.0001 (6)	0.0012 (6)	0.0020 (6)
Na2	0.0318 (8)	0.0238 (8)	0.0295 (9)	0.0042 (7)	0.0027 (7)	0.0019 (7)
Na3	0.0344 (9)	0.0290 (8)	0.0187 (8)	0.0055 (7)	0.0024 (6)	0.0036 (7)
Na4	0.0250 (7)	0.0195 (7)	0.0245 (8)	0.0035 (6)	0.0015 (6)	−0.0007 (6)
Na5	0.0280 (8)	0.0232 (7)	0.0219 (8)	0.0031 (6)	−0.0008 (6)	0.0017 (6)
O1	0.0271 (15)	0.0317 (16)	0.0252 (16)	0.0021 (13)	−0.0040 (13)	−0.0052 (13)
O2	0.0381 (17)	0.0225 (15)	0.0397 (19)	−0.0021 (13)	0.0135 (14)	−0.0004 (14)
O3	0.0264 (14)	0.0263 (14)	0.0193 (13)	0.0004 (11)	−0.0005 (11)	−0.0071 (11)
O4	0.0261 (14)	0.0174 (13)	0.0431 (18)	0.0043 (11)	−0.0029 (13)	−0.0030 (12)
O5	0.0380 (16)	0.0452 (18)	0.0170 (14)	−0.0123 (14)	0.0007 (12)	0.0077 (13)
O6	0.0236 (13)	0.0268 (14)	0.0242 (15)	0.0010 (11)	−0.0007 (11)	0.0086 (12)
O7	0.0265 (14)	0.0274 (14)	0.0209 (14)	0.0038 (11)	0.0048 (11)	−0.0024 (11)
O8	0.0241 (13)	0.0175 (12)	0.0273 (15)	−0.0027 (10)	−0.0028 (11)	0.0005 (11)
O9	0.0493 (19)	0.0403 (17)	0.0161 (14)	−0.0015 (15)	0.0045 (13)	0.0069 (13)
O10	0.0278 (15)	0.0198 (13)	0.0465 (19)	−0.0052 (12)	−0.0046 (13)	0.0022 (13)
O11	0.0306 (15)	0.0288 (14)	0.0205 (14)	0.0124 (12)	−0.0017 (11)	−0.0062 (12)
O12	0.0288 (15)	0.0237 (14)	0.0432 (19)	0.0058 (12)	0.0055 (13)	0.0122 (13)
O13	0.0287 (14)	0.0345 (16)	0.0207 (14)	0.0046 (12)	−0.0021 (11)	−0.0062 (12)
O14	0.0222 (13)	0.0282 (14)	0.0177 (13)	−0.0032 (11)	0.0024 (10)	0.0017 (11)
C1	0.0175 (17)	0.031 (2)	0.030 (2)	0.0028 (15)	0.0008 (15)	−0.0085 (17)
C2	0.030 (2)	0.035 (2)	0.032 (2)	−0.0031 (18)	−0.0025 (17)	−0.0074 (19)
C3	0.023 (2)	0.044 (3)	0.050 (3)	−0.0036 (19)	−0.0059 (19)	0.005 (2)
C4	0.040 (3)	0.091 (5)	0.069 (4)	−0.017 (3)	−0.016 (3)	−0.008 (4)
C5	0.0198 (17)	0.0196 (17)	0.026 (2)	0.0039 (14)	0.0010 (15)	0.0023 (15)
C6	0.025 (2)	0.0258 (19)	0.032 (2)	−0.0028 (16)	−0.0032 (16)	−0.0009 (17)
C7	0.0211 (19)	0.044 (3)	0.046 (3)	−0.0001 (19)	−0.0029 (18)	−0.004 (2)
C8	0.031 (3)	0.065 (4)	0.086 (5)	−0.014 (3)	−0.012 (3)	−0.004 (3)
C9	0.0201 (18)	0.034 (2)	0.0195 (19)	0.0016 (16)	−0.0022 (14)	−0.0025 (17)
C10	0.027 (2)	0.036 (2)	0.050 (3)	0.0024 (18)	0.0039 (19)	−0.010 (2)
C11	0.033 (2)	0.046 (3)	0.062 (3)	0.013 (2)	0.007 (2)	0.010 (3)
C12	0.032 (3)	0.058 (4)	0.120 (6)	0.013 (3)	0.005 (3)	−0.020 (4)
C13	0.0148 (17)	0.029 (2)	0.036 (2)	−0.0021 (15)	0.0011 (15)	0.0046 (18)
C14	0.0227 (19)	0.028 (2)	0.043 (3)	0.0036 (16)	−0.0029 (17)	0.0034 (19)
C15	0.023 (2)	0.045 (3)	0.045 (3)	0.0004 (19)	−0.0025 (18)	0.007 (2)
C16	0.027 (2)	0.051 (3)	0.051 (3)	0.010 (2)	−0.003 (2)	0.009 (2)

### Geometric parameters (Å, º)

**Table:**

Pd1—Cl1	2.2776 (12)	O1—H2	0.82 (3)
Pd1—Cl2	2.2800 (10)	O2—H3	0.81 (3)
Pd1—Cl3^i^	2.3159 (11)	O2—H4	0.80 (3)
Pd1—Cl3	2.3212 (12)	C1—C2	1.517 (6)
Cl2—Na1	2.8560 (18)	C1—H1A	0.9800
S1—O5	1.453 (3)	C1—H1B	0.9800
S1—O4	1.453 (3)	C2—C3	1.514 (6)
S1—O3	1.458 (3)	C2—H2A	0.9800
S1—C1	1.767 (4)	C2—H2B	0.9800
S2—O6	1.454 (3)	C3—C4	1.512 (8)
S2—O7	1.460 (3)	C3—H3A	0.9800
S2—O8	1.462 (3)	C3—H3B	0.9800
S2—C5	1.771 (4)	C4—H4A	0.9700
S3—O9	1.440 (3)	C4—H4B	0.9700
S3—O10	1.455 (3)	C4—H4C	0.9700
S3—O11	1.463 (3)	C5—C6	1.524 (5)
S3—C9	1.759 (4)	C5—H5A	0.9800
S4—O12	1.451 (3)	C5—H5B	0.9800
S4—O13	1.452 (3)	C6—C7	1.522 (6)
S4—O14	1.462 (3)	C6—H6A	0.9800
S4—C13	1.767 (4)	C6—H6B	0.9800
Na1—O6	2.284 (3)	C7—C8	1.520 (7)
Na1—O1	2.326 (3)	C7—H7A	0.9800
Na1—O11^ii^	2.386 (3)	C7—H7B	0.9800
Na1—O3	2.387 (3)	C8—H8A	0.9700
Na1—O8^iii^	2.540 (3)	C8—H8B	0.9700
Na2—O4^iv^	2.283 (3)	C8—H8C	0.9700
Na2—O1	2.407 (4)	C9—C10	1.516 (6)
Na2—O6^iii^	2.431 (3)	C9—H9A	0.9800
Na2—O2^iii^	2.488 (4)	C9—H9B	0.9800
Na2—O5^iii^	2.649 (3)	C10—C11	1.507 (7)
Na2—O8	2.700 (3)	C10—H10A	0.9800
Na3—O9	2.212 (3)	C10—H10B	0.9800
Na3—O5	2.264 (3)	C11—C12	1.514 (7)
Na3—O2	2.311 (4)	C11—H11A	0.9800
Na3—O13^v^	2.414 (3)	C11—H11B	0.9800
Na3—O7	2.649 (3)	C12—H12A	0.9700
Na4—O7	2.308 (3)	C12—H12B	0.9700
Na4—O12	2.342 (3)	C12—H12C	0.9700
Na4—O14^vi^	2.363 (3)	C13—C14	1.519 (6)
Na4—O10	2.393 (3)	C13—H13A	0.9800
Na4—O11^vii^	2.479 (3)	C13—H13B	0.9800
Na5—O8	2.391 (3)	C14—C15	1.529 (6)
Na5—O13	2.462 (3)	C14—H14A	0.9800
Na5—O3^iii^	2.465 (3)	C14—H14B	0.9800
Na5—O4^iv^	2.505 (3)	C15—C16	1.515 (6)
Na5—O11^vii^	2.582 (3)	C15—H15A	0.9800
Na5—O5^iv^	2.817 (4)	C15—H15B	0.9800
Na5—O10^vii^	2.931 (4)	C16—H16A	0.9700
Na5—O12	3.000 (4)	C16—H16B	0.9700
O1—H1	0.80 (3)	C16—H16C	0.9700
			
Cl1—Pd1—Cl2	92.45 (4)	O5^iv^—Na5—O12	119.93 (10)
Cl1—Pd1—Cl3^i^	90.99 (4)	O10^vii^—Na5—O12	76.60 (9)
Cl2—Pd1—Cl3^i^	176.53 (4)	Na1—O1—H1	108 (4)
Cl1—Pd1—Cl3	177.19 (4)	Na2—O1—H1	116 (4)
Cl2—Pd1—Cl3	90.36 (4)	Na1—O1—H2	109 (4)
Cl3^i^—Pd1—Cl3	86.20 (4)	Na2—O1—H2	107 (4)
Pd1—Cl2—Na1	139.61 (5)	H1—O1—H2	102 (5)
Pd1^i^—Cl3—Pd1	93.80 (4)	Na3—O2—Na2^iii^	88.93 (12)
O5—S1—O4	110.28 (19)	Na3—O2—H3	116 (4)
O5—S1—O3	111.62 (17)	Na2^iii^—O2—H3	122 (4)
O4—S1—O3	112.98 (17)	Na3—O2—H4	107 (4)
O5—S1—C1	108.38 (19)	Na2^iii^—O2—H4	117 (4)
O4—S1—C1	106.46 (18)	H3—O2—H4	104 (5)
O3—S1—C1	106.83 (17)	C2—C1—S1	114.3 (3)
O5—S1—Na5^v^	61.48 (14)	C2—C1—H1A	108.7
O6—S2—O7	110.06 (17)	S1—C1—H1A	108.7
O6—S2—O8	112.61 (17)	C2—C1—H1B	108.7
O7—S2—O8	112.35 (16)	S1—C1—H1B	108.7
O6—S2—C5	107.70 (17)	H1A—C1—H1B	107.6
O7—S2—C5	106.74 (17)	C3—C2—C1	112.1 (4)
O8—S2—C5	107.05 (17)	C3—C2—H2A	109.2
O9—S3—O10	112.86 (19)	C1—C2—H2A	109.2
O9—S3—O11	111.67 (18)	C3—C2—H2B	109.2
O10—S3—O11	110.37 (18)	C1—C2—H2B	109.2
O9—S3—C9	107.65 (19)	H2A—C2—H2B	107.9
O10—S3—C9	106.94 (19)	C4—C3—C2	112.3 (4)
O11—S3—C9	107.02 (18)	C4—C3—H3A	109.1
O12—S4—O13	111.59 (18)	C2—C3—H3A	109.1
O12—S4—O14	111.84 (17)	C4—C3—H3B	109.1
O13—S4—O14	112.33 (17)	C2—C3—H3B	109.1
O12—S4—C13	106.62 (18)	H3A—C3—H3B	107.9
O13—S4—C13	107.99 (19)	C3—C4—H4A	109.5
O14—S4—C13	106.06 (18)	C3—C4—H4B	109.5
O6—Na1—O1	90.30 (12)	H4A—C4—H4B	109.5
O6—Na1—O11^ii^	157.10 (12)	C3—C4—H4C	109.5
O1—Na1—O11^ii^	97.25 (12)	H4A—C4—H4C	109.5
O6—Na1—O3	95.34 (11)	H4B—C4—H4C	109.5
O1—Na1—O3	174.22 (12)	C6—C5—S2	111.9 (3)
O11^ii^—Na1—O3	78.00 (10)	C6—C5—H5A	109.2
O6—Na1—O8^iii^	80.06 (10)	S2—C5—H5A	109.2
O1—Na1—O8^iii^	97.50 (11)	C6—C5—H5B	109.2
O11^ii^—Na1—O8^iii^	77.54 (10)	S2—C5—H5B	109.2
O3—Na1—O8^iii^	84.77 (10)	H5A—C5—H5B	107.9
O6—Na1—Cl2	97.08 (9)	C7—C6—C5	112.6 (3)
O1—Na1—Cl2	81.71 (9)	C7—C6—H6A	109.1
O11^ii^—Na1—Cl2	105.37 (9)	C5—C6—H6A	109.1
O3—Na1—Cl2	96.29 (8)	C7—C6—H6B	109.1
O8^iii^—Na1—Cl2	177.04 (9)	C5—C6—H6B	109.1
O4^iv^—Na2—O1	114.31 (12)	H6A—C6—H6B	107.8
O4^iv^—Na2—O6^iii^	136.11 (12)	C6—C7—C8	111.1 (4)
O1—Na2—O6^iii^	100.08 (11)	C6—C7—H7A	109.4
O4^iv^—Na2—O2^iii^	103.31 (12)	C8—C7—H7A	109.4
O1—Na2—O2^iii^	76.94 (11)	C6—C7—H7B	109.4
O6^iii^—Na2—O2^iii^	110.63 (12)	C8—C7—H7B	109.4
O4^iv^—Na2—O5^iii^	87.44 (11)	H7A—C7—H7B	108.0
O1—Na2—O5^iii^	146.38 (12)	C7—C8—H8A	109.5
O6^iii^—Na2—O5^iii^	77.03 (11)	C7—C8—H8B	109.5
O2^iii^—Na2—O5^iii^	73.07 (11)	H8A—C8—H8B	109.5
O4^iv^—Na2—O8	74.15 (10)	C7—C8—H8C	109.5
O1—Na2—O8	98.82 (11)	H8A—C8—H8C	109.5
O6^iii^—Na2—O8	74.38 (10)	H8B—C8—H8C	109.5
O2^iii^—Na2—O8	173.81 (12)	C10—C9—S3	114.2 (3)
O5^iii^—Na2—O8	112.15 (10)	C10—C9—H9A	108.7
O9—Na3—O5	171.09 (14)	S3—C9—H9A	108.7
O9—Na3—O2	102.75 (13)	C10—C9—H9B	108.7
O5—Na3—O2	83.98 (13)	S3—C9—H9B	108.7
O9—Na3—O13^v^	90.64 (12)	H9A—C9—H9B	107.6
O5—Na3—O13^v^	83.01 (12)	C11—C10—C9	111.9 (4)
O2—Na3—O13^v^	93.81 (12)	C11—C10—H10A	109.2
O9—Na3—O7	85.12 (11)	C9—C10—H10A	109.2
O5—Na3—O7	102.30 (12)	C11—C10—H10B	109.2
O2—Na3—O7	76.45 (11)	C9—C10—H10B	109.2
O13^v^—Na3—O7	168.18 (11)	H10A—C10—H10B	107.9
O7—Na4—O12	93.62 (11)	C10—C11—C12	114.0 (5)
O7—Na4—O14^vi^	88.54 (10)	C10—C11—H11A	108.8
O12—Na4—O14^vi^	176.01 (12)	C12—C11—H11A	108.8
O7—Na4—O10	103.43 (11)	C10—C11—H11B	108.8
O12—Na4—O10	95.00 (12)	C12—C11—H11B	108.8
O14^vi^—Na4—O10	87.75 (11)	H11A—C11—H11B	107.7
O7—Na4—O11^vii^	98.26 (11)	C11—C12—H12A	109.5
O12—Na4—O11^vii^	86.19 (12)	C11—C12—H12B	109.5
O14^vi^—Na4—O11^vii^	90.18 (11)	H12A—C12—H12B	109.5
O10—Na4—O11^vii^	158.15 (12)	C11—C12—H12C	109.5
O8—Na5—O13	124.40 (12)	H12A—C12—H12C	109.5
O8—Na5—O3^iii^	86.35 (10)	H12B—C12—H12C	109.5
O13—Na5—O3^iii^	148.92 (12)	C14—C13—S4	114.4 (3)
O8—Na5—O4^iv^	76.20 (10)	C14—C13—H13A	108.7
O13—Na5—O4^iv^	109.44 (11)	S4—C13—H13A	108.7
O3^iii^—Na5—O4^iv^	79.97 (10)	C14—C13—H13B	108.7
O8—Na5—O11^vii^	76.62 (10)	S4—C13—H13B	108.7
O13—Na5—O11^vii^	107.11 (10)	H13A—C13—H13B	107.6
O3^iii^—Na5—O11^vii^	72.99 (9)	C13—C14—C15	111.9 (4)
O4^iv^—Na5—O11^vii^	142.52 (11)	C13—C14—H14A	109.2
O8—Na5—O5^iv^	127.94 (10)	C15—C14—H14A	109.2
O13—Na5—O5^iv^	71.62 (10)	C13—C14—H14B	109.2
O3^iii^—Na5—O5^iv^	93.85 (10)	C15—C14—H14B	109.2
O4^iv^—Na5—O5^iv^	52.85 (9)	H14A—C14—H14B	107.9
O11^vii^—Na5—O5^iv^	152.22 (10)	C16—C15—C14	113.4 (4)
O8—Na5—O10^vii^	127.13 (10)	C16—C15—H15A	108.9
O13—Na5—O10^vii^	72.78 (9)	C14—C15—H15A	108.9
O3^iii^—Na5—O10^vii^	85.23 (10)	C16—C15—H15B	108.9
O4^iv^—Na5—O10^vii^	151.57 (11)	C14—C15—H15B	108.9
O11^vii^—Na5—O10^vii^	51.04 (8)	H15A—C15—H15B	107.7
O5^iv^—Na5—O10^vii^	104.68 (9)	C15—C16—H16A	109.5
O8—Na5—O12	81.05 (10)	C15—C16—H16B	109.5
O13—Na5—O12	51.00 (9)	H16A—C16—H16B	109.5
O3^iii^—Na5—O12	144.57 (10)	C15—C16—H16C	109.5
O4^iv^—Na5—O12	127.80 (10)	H16A—C16—H16C	109.5
O11^vii^—Na5—O12	71.92 (9)	H16B—C16—H16C	109.5

Symmetry codes: (i) −*x*, −*y*+1, −*z*+1; (ii) *x*, −*y*+1/2, *z*+1/2; (iii) −*x*+1, −*y*+1, −*z*+1; (iv) *x*, *y*+1, *z*; (v) *x*, *y*−1, *z*; (vi) −*x*+1, *y*−1/2, −*z*+1/2; (vii) −*x*+1, *y*+1/2, −*z*+1/2.

### Hydrogen-bond geometry (Å, º)

**Table:**

*D*—H···*A*	*D*—H	H···*A*	*D*···*A*	*D*—H···*A*
O1—H1···O14^viii^	0.80 (3)	2.10 (4)	2.879 (4)	164 (5)
O1—H2···Cl1	0.82 (3)	2.32 (4)	3.127 (3)	172 (6)
O2—H3···O10^vi^	0.81 (3)	1.95 (4)	2.723 (4)	159 (5)
O2—H4···O14^vi^	0.80 (3)	2.10 (4)	2.884 (4)	164 (6)

Symmetry codes: (vi) −*x*+1, *y*−1/2, −*z*+1/2; (viii) *x*, −*y*+3/2, *z*+1/2.
